# Encapsulated *Escherichia coli *in alginate beads capable of secreting a heterologous pectin lyase

**DOI:** 10.1186/1475-2859-4-35

**Published:** 2005-12-14

**Authors:** Rigini M Papi, Sotiria A Chaitidou, Fotini A Trikka, Dimitrios A Kyriakidis

**Affiliations:** 1 Laboratory of Biochemistry, Department of Chemistry, Aristotle University of Thessaloniki, Thessaloniki, 54124, Greece; 2 National Hellenic Research Foundation, 48 Vasileos Konstantinou Ave., 11635 Athens, Greece

## Abstract

**Background:**

Production of heterologous proteins in the *E. coli *periplasm, or into the extracellular fluid has many advantages; therefore naturally occurring signal peptides are selected for proteins translocation. The aim of this study was the production in high yields of a recombinant pectin lyase that is efficiently secreted and the encapsulation of transformed *E. coli *cells for pectin degradation in a biotechnological process.

**Results:**

The nucleotide sequence of *Bacillus subtilis *α-amylase's signal peptide was fused to the N-terminal of an heterologously expressed pectin lyase in *E. coli *BL21 [DE3]. Thus pectin lyase secretion was achieved into the extracellular growth medium. *E. coli *cells harboring the recombinant plasmid heterologously express pectin lyase to around 22% of the total cellular proteins, as it was estimated by SDS-PAGE and image analysis. IPTG induces the heterologously expressed enzyme, which is initially distributed extracellularly (7 hour) and later on at the periplasmic (9 hours) or cytosolic fraction (20 hours). No pectin lyase activity was found in the membranes fraction and in the inclusion bodies. Encapsulation of the recombinant strains of *E. coli *in alginate or alginate/silica beads 1:5 showed that pectin lyase could degrade effectively its substrate, for at least ten operational cycles.

**Conclusion:**

Secretion of an heterologously overexpressed pectin lyase in *E. coli *BL21 [DE3] was achieved in this study. For this purpose the signal peptide of α-amylase from *B. subtilis *was fused to the N-terminal domain of pectin lyase. Encapsulated *E. coli *BL21 [DE3] cells harboring pET29c/exPNL were used successfully for pectin degradation up to ten operational cycles indicating that under special conditions this might have biotechnological implementations.

## Background

Pectin lyase (PNL, EC 4.2.2.10) belongs to polysaccharide lyase family 1 and cleaves α-1,4 bonds between galacturonic acid residues of pectin. The mode of PNL action is trans-elimination [1] with end-product oligosaccharides of 4-deoxy-6-methyl-alpha-D-galact-4-enuronosyl groups. Pectins are present in the primary cell wall and intracellular space of higher plants. The enzymic degradation of pectin is of great interest in food industry for fruit juice clarification, reduction of turbidity and increase of the amount of juice extracted from fruit pulp [2,3]. The enzymic degradation is also used in the textile industry in the processing of natural fibers [4]. Among the pectinolytic enzymes, pectin lyase is the only one known to degrade highly esterified pectin without the previous action of other enzymes [5].

Heterologous expression of pectin lyase gene (*pnl*) from *Pseudomonas marginalis *N6301 in *E. coli *BL21 [DE3] has previously been reported [6]. The enzyme was overproduced in the cytoplasm, bearing a 6-His-tag at the C-terminal, and it was purified close to homogeneity by affinity chromatography on a Ni^++^-NTA agarose column. Even though the heterologous expression of PNL was achieved in high yields there was a number of limitations compared to PNL production from its native source, the bacterium *P. marginalis*. It was necessary to disrupt bacterial cells to recover the enzyme with the proper protein folding.

Since secretion of recombinant proteins is preferred, many signal sequences derived from naturally occurring secretory proteins could be selected to support the efficient translocation of this heterologous polypeptide across the inner membrane, when fused to its amino termini [7]. Signal peptides usually consist of a positively charged amino-terminus (n-region, 1–5 residues in length), a central hydrophobic domain (h-region, 7–15 residues) and a neutral but polar carboxy-terminus domain (c-region, 3–7 residues). The c-region specifies the signal peptide cleavage site for specific signal peptidases whereas the other two domains are required for efficient translocation [8]. The length of signal peptides ranges from 22–32 amino acids. The majority of *E. coli *secreted proteins is either localized in the periplasm, or is associated with the inner or outer membranes [9]. Although recombinant proteins targeted to the periplasm remain to this cellular compartment, their release into the extracellular fluid may occur through non-specific leakage, or due to cell lysis [10,11].

The signal peptide of α-amylase from the gram-positive bacterium *Bacillus subtilis *cloned and excreted in *E. coli*, a Gram^- ^bacterium, maps among the signal peptides of *E. coli *excreted proteins [12]. This *Bacillus *signal peptide can also achieve secretion in *E. coli *of the periplasmic space protein β-lactamase to the extracellular surroundings [13].

In this report, we studied the fusion of the 33-aminoacid-signal sequence of *B. subtilis *α-amylase to the amino-terminal domain of pectin lyase and the secretion of the heterologous expressed pectin lyase. The transformed bacteria were further encapsulated in alginate beads and were tested successfully for pectin degradation.

## Results

### Construction of a himeric PNL carrying the signal peptide of *B. subtilis *α-amylase

**Figure 1:**
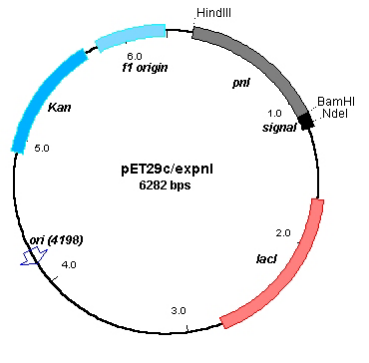
Plasmid diagram of pET29c/*expnl*. Genes and major features of the plasmid are indicated by thick boxes. The restriction sites used in the construction are indicated as well.

Isolated genomic DNA from *B. subtilis *was used as template for the amplification of the signal sequence of α-amylase gene. Since the desired signal sequence of α-amylase was very small, various primers were designed to anneal upstream and downstream of the promoter and the coding region of α-amylase. After electrophoresis of PCR products on 1.5% w/v agarose gel the amplification fragment of the appropriate size was used as DNA template in a second PCR with primers only for the signal sequence of α-amylase. Amplification of α-amylase signal sequence was confirmed by agarose gel electrophoresis. Fragments were extracted from agarose gel with QIAEXII Gel Extraction Kit (Qiagen) and both signal sequence and pET29c/*pnl *were digested with NdeI and BamHI. After ligation of the digested plasmid and the insert, plasmid pET29c/*expnl *was generated (Fig. 1). The existence of α-amylase's signal sequence in pET29c/*expnl *was confirmed by DNA sequencing.

### Overexpression of exPNL

**Figure 2:**
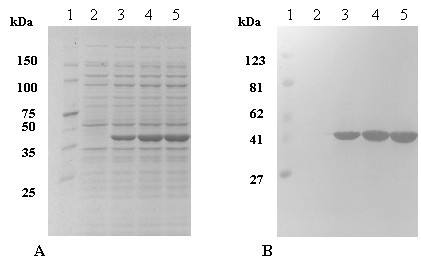
Overexpression of *expnl *gene. **A: **SDS-PAGE of *E. coli *exPNL total cells, lane 1: pre-stained protein markers, lane 2: before the induction, lane 3: 1 h after the induction, lane 4: 2 h after the induction, lane 5: 3 h after the induction. Gel was stained with Coomassie Brilliant Blue R-250 **B: **transfer of proteins electrophoresed as in A and immunostained with anti-His (1:500)

Cultures of *E. coli *BL21 [DE3] harboring pET29c/*expnl *were cultivated in LB medium and ex*pnl *gene was overexpressed after induction with IPTG. The total expressed proteins are shown at Fig. 2A. Overexpression of exPNL appears at lanes 3–5 of Fig. 2A. The presence of the recombinant exPNL was also confirmed by immunostaining of the same gel by anti-6-His (1:500 dilution), as shown at Fig. 2B lanes 3–5. The overproduction of exPNL was around 22% of the total amount of proteins, as estimated by the Gel-Pro Analyzer (version 3, Media Cybemetics, 1993–97) with the expected molecular mass of 39 kDa.

### Subcellular distribution of exPNL activity

**Figure 3:**
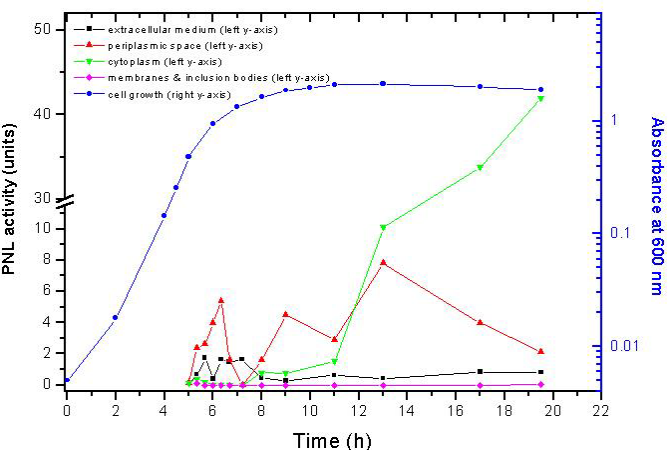
The appearance of exPNL activity in different fractions of *E. coli*, transformed with pET29c/*expnl*, during bacterial growth.

**Figure 4:**
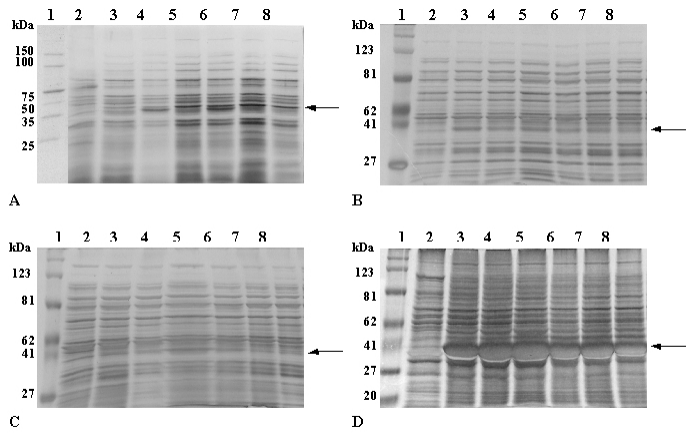
SDS-PAGE of the subcellular fractions of *E. coli *transformed with pET29c/*expnl*, at different times of cell growth in presence of IPTG. **A: **extracellular medium, **B: **periplasmic space, **C: **cytoplasm, **D: **membranes and inclusion bodies – Lane 1: protein markers, lane 2: before the induction with IPTG, lane 3: 20 min after the induction, lane 4: 40 min after the induction, lane 5: 1 h after the induction, lane 6: 1 h & 40 min after the induction, lane 7: 3 h after the induction, lane 8: 6 h after the induction. Gels were stained with silver nitrate.

The appearance of pectin lyase in the extracellular fluid as well as at different subcellular fractions was studied. The presence of exPNL was estimated both by following its activity (Fig 3) and by SDS-PAGE (Fig. 4). In the periplasmic fraction exPNL activity was induced right after the addition of IPTG, with a peak at the first hour of induction. One hour later exPNL activity dramatically declined and then another peak of activity appeared 4–8 hours after IPTG addition. An increment of exPNL activity in the extracellular fluid was observed with a small delay comparing to that in the periplasm.

**Figure 5:**
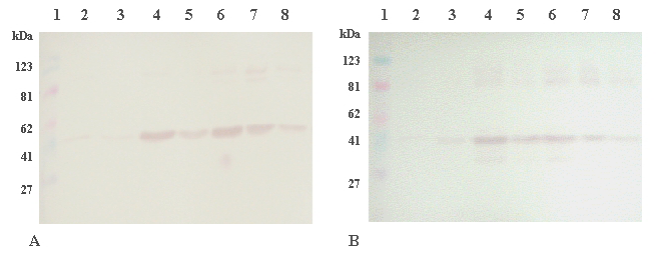
Total proteins electrophoresed as described at figure 5A, transferred and immunostained with anti-six-His (A) and anti-PNL (B).

In the cytosolic fraction exPNL activity was increased 4 hours after induction with IPTG, whereas activity was not detectable in other fractions. Total protein (10 μg) of each subcellular fraction and the extracellular medium, were electrophoresed and the proteins were stained with silver nitrate (Fig. 4). A large amount (12%) of inactive exPNL was present in the 100,000 × g pellet, as inclusion bodies, as it was judged by measuring the stained protein by the Gel-Pro Analyzer (Fig. 4D). Solubilization of inclusion bodies with 8 M urea or 6 M guanidine hydrochloride and renaturation by dialysis as described in Material and Methods resulted in an inactive enzyme. The extracellularly secreted exPNL was also immunostained with antiHis (1:500 dilution) and antiPNL (1:500 dilution) (Fig. 5). These results indicate that exPNL is overproduced by IPTG induction and migrates to the periplasmic fraction and is finally excreted to the extracellular medium.

### Cell encapsulation in alginate beads

**Figure 6:**
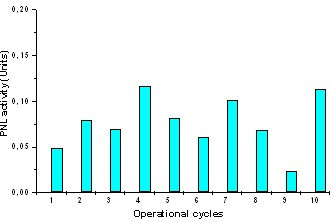
Enzymic degradation of pectin by encapsulated in alginate beads *E. coli *cells transformed with pET29c/*expnl*. Each column represents pectin lyase activity measured at 20 min of incubation.

Sodium alginate is soluble in water and becomes a hydrogel in the presence of multivalent cations (Ca^2+^, Ba^2+^, Sr^2+^). The resultant gel shows good permeability for small molecules [14]. In addition, the gelation of alginate can be achieved under mild conditions for immobilizing living cells. Thus, a variety of living cell phenotypes has been enclosed in such matrices [15-17]. To use more successfully the induced exPNL, transformed bacteria were encapsulated in alginate beads and used to degrade pectin for at least 10 operational cycles. Figure 6 shows the liberated exPNL activity in the extracellular fluid of the encapsulated bacteria at different operational cycle. After each operational cycle the encapsulated cells were allowed to rest for 1 hour in LB medium at 37°C. The pattern of liberated exPNL activity in the extracellular fluid is very similar for the repeated batches, reaching maximal exPNL activity at 20 minutes of incubation. Even though the activity of exPNL during the first cycle was very small, in all other cycles it was very sufficient. As figure 6 shows a repetitive motif of increase and decrease of enzymic activity is observed that may reflect the cell growth inside the beads. Furthermore, the addition of toluene at concentrations of 0.2 mg/ml and 0.4 mg/ml in the reaction mixture resulted, in both cases, in the increase of cell membrane permeability and finally in the 55% increase of liberated exPNL by the encapsulated cells.

## Discussion

There is a large number of reports on the secretory expression of recombinant proteins from *E. coli *[9,18]. Secreted polypeptides are synthesized as preproteins containing an amino-terminal signal sequence that is cleaved during the translocation process. This signal sequence is recognized and then cleaved by inner-membrane-associated signal peptidases. The secretion efficiency of these proteins is strongly dependent on the signal sequence used, the overall structure of the precursor protein and the processing of the signal peptide. Periplasmic production of recombinant proteins has several advantages with respect to cytoplasmic production, but frequently overproduced foreign proteins are not efficiently secreted to the periplasm in *E. coli*.

According to Sjöström et al. [12] the signal peptide of *B. subtilis *α-amylase maps among the signal peptides of *E. coli *excreted proteins and when it was added to β-lactamase the protein was secreted to the periplasm [13]. It is also stated that the secretion mechanism of *B. subtilis *is similar to that of *E. coli *with minor differences with respect to substrate specificities of their signal peptidases, or in other proteins related to secretion. In particular, the signal peptide of α-amylase has a sequence of Ala-Ala-Ser-Ala recognized by type I signal peptidases, which are common in *B. subtilis *and *E. coli *[19]. These data lead us to the construction of plasmid pET29c/*expnl *from pET29c/*pnl *carrying the *B. subtilis *α-amylase's signal sequence, introduced by NdeI and BamHI restriction sites (Fig. 1).

The recombinant PNL was secreted in the extracellular medium at around 30% of the total activity at the early stage of induction and later on the activity accumulated in the cytoplasm. Those results suggested that either the bacterial secretory mechanism was not working effectively, or that the degradation of the protein was very fast.

Although the transformed bacteria were cultivated at low temperature, the overproduced exPNL was partially segregated as inclusion bodies. Recent reports presented that co-overproduction of SecB, DnaK-DnaJ and GroEL-GroES has met variable success and improved secretion for a number of heterologous proteins in *E. coli *[20-23]. Since the signal sequence influences secondary and tertiary structure formation of the secretory proteins, which in turn affect chaperone recognition, we would like to try several signal sequences and/or overproduce different chaperones to optimize the translocation of the overproduced exPNL in the near future. Even though exPNL was not secreted in high levels and the rate of hydrolysis was low, in our experiments we showed that transformed *E. coli *with pET29/*expnl *encapsulated in alginate beads or alginate/silica gel 1:5 can be used for pectin degradation effectively, for at least ten operational cycles.

So far the commercial pectolytic preparations that are used in food processing industries, are crude multi-enzyme preparations from *Aspergillus niger *and contain hemicellulase, cellulase, xylanase, pectin esterase, polygalacturonase and pectin lyase [24]. Although pectolytic enzymes are widely used for industrial purposes, little research has been carried out on pectolytic enzymes immobilized in solid matrices and even less on cell encapsulation. Previously pectin lyase has been immobilized on DEAE-cellulose, on porous glass [25] and recently on nylon, with no loss of enzymic activity after 12 cycles of operation [26]. Our results of using encapsulated bacterial cells capable of secreting pectin lyase appear to be of potential interest for an industrial application.

## Conclusion

The aim of this study was the secretion of an heterologously overexpressed pectin lyase in *E. coli *BL21 [DE3]. For this purpose, the signal peptide of α-amylase from *B. subtilis *was fused to the N-terminal domain of pectin lyase that had been previously [6] cloned and overexpressed in *E. coli *BL21 [DE3]. Secretion into the extracellular medium was observed right after IPTG induction but then the fusion protein was accumulated in the periplasm and cytoplasm. Encapsulated *E. coli *BL21 cells harboring pET29c/exPNL in alginate beads, capable of secreting pectin lyase were used successfully for pectin degradation up to ten operational cycles, providing expenditure for future biotechnological applications.

## Materials and methods

### Material

All chemical compounds were purchased from SIGMA, Fluka, Baker and BDH Chemicals. Restriction enzymes, *Taq *polymerase and other reagents needed for molecular biology experiments were purchased from New England Biolabs.

### Isolation of DNA

Genomic DNA from *B. subtilis *was isolated according to Saito and Miura as modified by Gay [27,28]. Plasmid DNA was extracted by alkaline lysis [29].

### Construction of pET29c/*expnl *containing α-amylase's signal sequence

We have recently reported that *pnl *gene of *P. marginalis *was amplified by PCR and cloned into plasmid pET29c using BamHI and HindIII restriction sites [6]. In this paper plasmid pET29c/*expnl *is constructed from pET29c/*pnl *and *B. subtilis *α-amylase's signal sequence [30] to secrete pectin lyase. Larger fragments containing α-amylase's signal sequence were amplified by PCR using *B. subtilis *genomic DNA as the template and proper oligonucleotides. The reaction was performed as follows: 50 μl reaction volume was prepared to contain 2 units of *Taq *polymerase, 5 μl of polymerase buffer, 200 μM of each dNTP, 0.13 μg/μl of each primer and 1 ng of template DNA. The reaction mixture was cycled 30 times using the following conditions: 92°C for 1 min, 54°C for 30 sec, 72°C for 50 sec and another 5 min at 72°C at the end of the cycles. The amplification products were electrophoresed in 1.5% w/v agarose gel and products of right size were used as the template DNA in a second PCR, where primers specific for α-amylase's signal sequence and with NdeI and BamHI restriction sites were used (forward: 5'-GGAATTCCATATGTTTGCAAAACGA-3', reverse: 5'-CGCGGATCCTACTCGCAGCC-3'). The reaction mixture and the conditions for PCR were the same as described above. The PCR product was electrophoresed in 1.5% w/v agarose gel and then was cut and extracted from the gel using the Qiagen gel extraction kit. The 99 bp NdeI-BamHI PCR fragment was introduced into the NdeI-BamHI fragment of pET29c/*pnl *resulting in pET29c/*expnl *plasmid. *E. coli *BL21 [DE3] competent cells were prepared according to Sambrook et al. [29] and transformed with pET29c/*expnl*.

### Microorganisms and growth conditions

The recombinant strains of *E. coli *BL21 [DE3] with the constructed plasmid pET29c/*expnl *were grown at 37°C, in LB medium containing kanamycin (50 μg/ml) with vigorous agitation. When cells reached an optical density of 0.6 at 600 nm, IPTG (1 mM) was added and the cultures were incubated at 24°C, for 3 hours. *B. subtilis *was grown at 37°C, in LB medium with vigorous agitation.

### Preparation of cell extracts and subcellular fractionation

Recombinant strains of *E. coli *BL21 [DE3] harboring pET29c/*expnl*, grown as described above, were harvested by centrifugation at 3,000 × g for 15 min at 4°C. The cell pellet was washed twice with PBS and suspended in 5 × ml of 0.5 M sucrose, 250 mM EDTA and 2 mg/ml lysozyme. The suspension was incubated on ice for 30 min and centrifuged at 20,000 × g for 15 min. The supernatant was kept/saved as the periplasmic fraction. The remaining pellet was suspended in 50 mM Tris-HCI (pH 7.5), 2.5 mM EDTA and then sonicated (5 times × 30 sec, 0.5 cycle, 50% amplitude – UP200 s dr. hielscher, GmbH). After addition of MgSO_4 _to a final concentration 40 mM, the mixture was centrifuged at 100,000 × g for 40 min. The 100,000 × g supernatant was kept as the cytoplasmic fraction and the 100,000 × g pellet as the membrane fraction with the inclusion bodies. The pellet 100,000 × g was suspended in 1 ml of 50 mM Tris-HCl buffer pH 7.5 [31].

### Enzyme assay

PNL activity was determined spectrophotometrically by monitoring the increase in absorbance at 235 nm of a solution containing 0.1% w/v pectin (Sigma, P-9135) in 50 mM citrate/phosphate buffer pH 6.5 at 37°C. The increase in absorbance was measured after 20 minutes of incubation. One unit of PNL activity was defined as the nmoles of unsaturated oligogalacturonides produced per minute, under the above-specified condition [32]. Specific activity of PNL was defined as the ratio of PNL units per mg of protein extract. The absorption coefficient of unsaturated oligogalacturonides is 5500 M^-1^cm^-1^.

### Electrophoresis and immunoblotting

SDS electrophoresis was performed in 10% w/v polyacrylamide gels containing 0.1% w/v SDS as described by Laemmli [33]. Protein content was determined by the method of Bradford [34]. Gels were stained either with Coomassie Brilliant Blue R250 [35] or with silver nitrate [36]. Proteins were transferred to nitrocellulose membranes following the method of Towbin et al. [37] and immunostained as described [38].

### Solubilization and renaturation of inclusion bodies

The 100,000 × g pellet containing the inclusion bodies was either dissolved in 10 fold volume of 50 mM Tris-HCl pH 8.0, 5 mM NaCl, 5 mM MgCl_2_, 2.5 mM β-MSH, 0.1% w/v Tween 20 (DR, Denaturation – Renaturation buffer) containing 8 M urea, or in 50 mM Tris-HCl pH 8.0, 6 M guanidine hydrochloride, 1 mM DTT to a final concentration of 1 mg/ml. Solubilization of inclusion bodies was proceed at room temperature for 1 hour. In the first case the mixture was centrifuged at 100,000 × g for 20 min at 4°C and then the 100,000 × g supernate was dialyzed gradually against DR buffer containing lower concentrations of urea (6, 4, 2, 1 mM urea) and finally overnight just in DR buffer. In the second case the mixture was diluted tenfold with the same buffer and dialyzed overnight against a 100 fold volume of 50 mM Tris-HCl pH 8.0, 150 mM NaCl, 1 mM DTT and 5% glycerol. Dialysis was followed by centrifugation at 50,000 × g for 30 min at 4°C and enzyme activity was determined. In all steps 1 mM PMSF was added.

### Formation of alginate beads

After 2 hours of induction with IPTG, cells were harvested at 3000 × g for 20 min and mixed with sterile water (300 mg cells/ml). Sodium alginate solution (2% v/v) was mixed with the above cell suspension at a volumetric ratio of 9:1 and the mixture was dropped with an insulin syringe into a gently stirred 1% w/v solution of BaCl_2 _at a volumetric ratio of 1:50. A spontaneous cross-linking reaction produced spherical hydrogel beads of barium alginate, which remained for 30 minutes at 4°C in order to stabilize the formed network. After stabilization, the beads were washed with 0.9% v/v NaCl (twice) and used immediately. All solutions that were used are sterile.

### Pectin hydrolysis by the transformed bacteria encapsulated in alginate beads

Pectin hydrolysis was performed in conical flasks at 37°C under gentle stirring and each operational cycle lasts for 1 h. The substrate used was the same as for enzyme assay with the addition of LB solution (25:1 final concentration) and BaCl_2 _at a final concentration of 0.005% v/v in order to preserve cell viability and network stability. At the end of each cycle, beads were washed with 0.9% v/v NaCl (twice) and placed with fresh LB solution at room temperature under gentle shaking for 1 h. This procedure was repeated after each operational cycle.

## Abbreviations

PNL, pectin lyase; exPNL, extracellular pectin lyase; IPTG, isopropylthiogalactoside

## Authors' contributions

All authors contributed equally in this paper.
